# Predicting stroke and death in patients with heart failure using CHA_2_DS_2_-VASc score in Asia

**DOI:** 10.1186/s12872-019-1178-0

**Published:** 2019-08-08

**Authors:** Mi Kyoung Son, Nam-Kyoo Lim, Hyun-Young Park

**Affiliations:** 1 Division for Cardiovascular Diseases, Korea National Institute of Health, 187 OsongSaengmyeong2-Ro, Osong-Eup, Cheongju, Chungcheongbuk-Do Republic of Korea; 2 Center for Genome Science, Korea National Institute of Health, 187 OsongSaengmyeong2-Ro, Osong-Eup, Cheongju, Chungcheongbuk-Do Republic of Korea

**Keywords:** Heart failure, Stroke, Atrial fibrillation, CHA_2_DS_2_-VASc score

## Abstract

**Background:**

The CHA_2_DS_2_-VASc score is used to assess risk of mortality as well as to stratify risk of stroke in patients with atrial fibrillation (AF). This study evaluated whether CHA_2_DS_2_-VASc score was predictive of 1 and 2 year risks of stroke and death in Asian patients with heart failure (HF).

**Methods:**

Patients hospitalized for HF were enrolled in the Korean Acute Heart Failure (KorAHF) registry, a prospective observational multicenter cohort study, between March 2011 and February 2014. Patients with a history of cancer before hospitalization for HF were excluded. The discriminatory properties of the CHA_2_DS_2_-VASc score were quantified using C-statistics.

**Results:**

The study included 5158 patients with HF, 2091 with and 3067 without AF. Rates of stroke in these two groups were 4.5 and 2.8%, respectively, after 1 year, and 5.5 and 3.4%, respectively, after 2 years. Each 1-point increase in CHA_2_DS_2_-VASc score was associated with significantly increased risks of stroke and all-cause death in HF patients with and without AF (*p*-value < 0.05). The C-statistics of the CHA_2_DS_2_-VASc score for all-cause death in patients with and without AF were 0.600 and 0.630, respectively, at 1 year and 0.626 and 0.635, respectively, at 2 years. The C-statistics for stroke ranged from 0.593 to 0.639.

**Conclusions:**

Among patients with incident HF with and without AF, CHA_2_DS_2_-VASc score was significantly associated with the risks of stroke and death. However, CHA_2_DS_2_-VASc score was only a modest predictor of stroke and death, indicating the need for studies evaluating modified CHA_2_DS_2_-VASc scores. The majority of strokes occurred relatively shortly after hospitalization for HF and that mortality rates in patients with HF remain high. Thus, early treatment after HF to prevent stroke is essential.

**Electronic supplementary material:**

The online version of this article (10.1186/s12872-019-1178-0) contains supplementary material, which is available to authorized users.

## Background

Heart failure (HF) occurs when the heart is unable to supply sufficient blood to the body, due to a structural and/or functional cardiac abnormality, resulting in reduced cardiac output and/or elevated intracardiac pressures at rest or during stress [1]. Atrial fibrillation (AF) is the most common sustained arrhythmia observed in clinical practice [2], and has been associated with a 5-fold higher risk of stroke compared with the general population [3]. The risk of ischemic stroke within 1 month after a diagnosis of HF was also found to be increased more than 5-fold [4], indicating that both HF and AF are associated with increased risks of ischemic stroke and mortality [3–7].

The CHA_2_DS_2_-VASc score is calculated from various factors, including congestive HF, hypertension, age ≥ 75 years (doubled risk), diabetes, stroke/transient ischemic attack/thromboembolism (doubled), vascular disease (prior myocardial infarction, peripheral artery disease, or aortic plaque), age 65–74 years, and female sex [8]. This simple clinical risk score is commonly used to stratify the risk of stroke in patients with AF [9].

The clinical utility of the CHA_2_DS_2_-VASc score in predicting the risks of ischemic stroke, thromboembolism, and death has extended beyond subjects without AF [10, 11]. For example, the CHA_2_DS_2_-VASc score was shown to predict the risks of ischemic stroke, thromboembolism, and death in patients with incident HF with or without AF [12]. Moreover, the predictive accuracy of the CHA_2_DS_2_-VASc score for death and stroke was reported to be modest in patients with systolic HF in sinus rhythm [13]. These findings suggested that CHA_2_DS_2_-VASc score can be used to stratify the risks of ischemic stroke and death in patients with HF. However, its predictive accuracy in Asian patients with HF has not yet been determined. Because the risks for stroke and death in patients with HF vary by racial/ethnic groups, it is necessary to assess the performance of the CHA_2_DS_2_-VASc score in Asian patients with HF [4, 5, 12, 13]. This study therefore evaluated whether CHA_2_DS_2_-VASc predicts stroke and death in patients with HF, with and without AF, in the Korean Acute Heart Failure (KorAHF) registry.

## Methods

### Data source

This study utilized the KorAHF registry, an ongoing, prospective observational multicenter cohort study, that has enrolled patients hospitalized for acute HF at 10 tertiary university hospitals across the country from March 2011 to February 2014 [14, 15]. This registry consists of 5625 patients diagnosed with HF by clinician and either (i) lung congestion, defined as “congestion” on chest X-rays or as rales on physical examination, or (ii) objective findings of left ventricular (LV) systolic dysfunction or structural heart disease. Real-time administrative data were collected using the Internet-based Clinical Research and Trial Management System (iCReaT), supported by the Korean National Institute of Health (KNIH). Information on a patient’s demographic characteristics, eligibility evaluation, clinical history, symptoms, physical measurements, electrocardiographic findings, medications, additional treatments, complications, and outcomes were documented at admission and during the follow-up. The study design, data validation, and interim analysis of this registry have been reported [14, 15]. The study protocol was approved by the Institutional Review Board of each hospital. Written informed consent was obtained from each patient, or from a relative or legal representative. All patients, except death, were followed up for at least 2 years, and data were collected through November 30, 2016.

### Study population

**Fig. 1:**
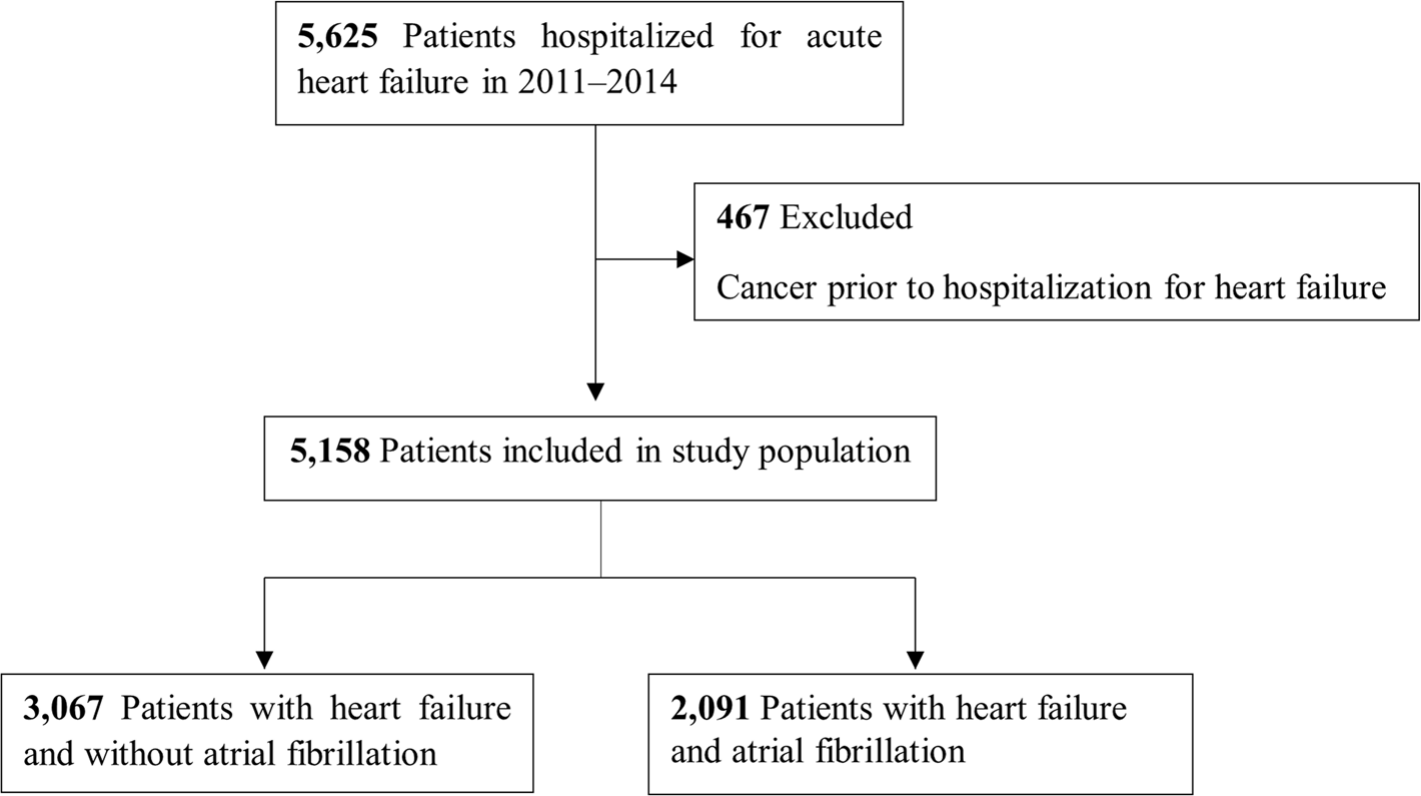
Selection of study population

Of 5625 patients hospitalized for HF, 467 with a history of cancer before hospitalization for HF were excluded. Thus, the study cohort consisted of 5158 patients with HF, 2091 with and 3067 without AF (Fig. 1).

The CHA_2_DS_2_-VASc score for each patient was calculated as described [8]. Patients with HF only and no additional risk factors for stroke were assigned a CHA_2_DS_2_-VASc score of 1. The CHA_2_DS_2_-VASc score was considered a categorical variable, classified as scores of 1, 2, 3, 4, 5, 6, and ≥ 7, to calculate the incidence rates for stroke and death. The CHA_2_DS_2_-VASc score was also considered a continuous variable to assess its ability to predict stroke and death. Medications used before hospital discharge were analyzed. Information on stroke and all-cause mortality was recorded during follow-up.

### Statistical analysis

Baseline characteristics at the time of hospitalization for HF were described using means and standard deviations for continuous variables and percentages for categorical variables. The rates of stroke and all-cause death were computed as the number of patients with stroke and death during the follow-up period, divided by the total person-years at risk, and were reported as number of patients per 100 person-years.

The association between CHA_2_DS_2_-VASc score and each outcome was determined by Cox’s proportional hazard regression analyses, performed after adjusting for potential confounders. Hazard ratios (HRs) and 95% confidence intervals (CIs) were estimated. Also, competing risk regression analysis using the Fine and Gray model was performed, taking into account the high mortality rate of patients with acute HF [16]. The discriminatory properties of the CHA_2_DS_2_-VASc score were quantified using the area under the receiver-operating characteristic (ROC) curve (AUC) and time-dependent AUC [17]. The AUC and 95% CI for each endpoint were calculated by non-parametric methods. Time-dependent AUC was considered the probability that a randomly selected patient who experienced the event of interest before a given time had a higher risk score than a control patient who did not experience that event before a given time. Time-dependent AUC was calculated for 1 and 2 year follow-up, and their 95% CIs were obtained by bootstrapping using 1000 bootstrap samples.

All statistical tests were two-tailed, and *p*-values of < 0.05 were considered statistically significant. All statistical analyses were performed using SAS software (ver. 9.4; SAS Institute, Cary, NC, USA) and R version 3.6.1 (R Foundation for Statistical Computing) with the timeROC and crr packages [18, 19].

## Results

The baseline characteristics of the study participants, with and without AF, are presented in Table 1. Most patients were older than 60 years of age (75.7%) and had hypertension (62.6%). Compared with patients with HF alone, those with HF and AF were older and significantly more likely to have had a previous stroke (19.6% vs. 12.4%), valvular heart disease (VHD) (29.1% vs. 12.5%), and chronic obstructive pulmonary disease (COPD) (12.0% vs. 10.1%); to be a never smoker (65.5% vs. 58.0%); and to have been treated with an aldosterone antagonist (59.1% vs. 53.9%), loop diuretics (94.0% vs. 89.9%), digoxin (54.6% vs. 18.5%), and warfarin (60.6% vs. 13.1%). By contrast, the HF patients without AF were more likely to have diabetes mellitus (44.4% vs. 34.4%), previous chronic renal failure (15.4% vs. 12.4%), ischemic heart disease (IHD) (52.5% vs. 30.0%), and cardiomyopathy (29.3% vs. 24.3%); to be a current smoker (22.0% vs. 13.2%); and to have been treated with angiotensin-converting enzyme inhibitors (41.3% vs. 34.1%), angiotensin receptor blockers (47.1% vs. 44.3%), β-blockers (59.7% vs. 56.5%), aspirin (70.7% vs. 57.1%), and statins (53.9% vs. 37.3%).

**Table 1:** Baseline characteristics of the study population, stratified according to diagnosis of atrial fibrillation

Characteristics	No. (%) of patients			*P*-value
	Overall (*n* = 5158)	With AF (*n* = 2091)	Without AF (*n* = 3067)	
Female	2416 (46.8)	1025 (49.0)	1391 (45.4)	0.010
Age, mean (SD), years	68.4 (14.7)	70.7 (12.6)	66.8 (15.7)	< 0.001
Age group, years				< 0.001
< 40	271 (5.3)	46 (2.2)	225 (7.3)	
40–49	341 (6.6)	99 (4.7)	242 (7.9)	
50–59	638 (12.4)	236 (11.3)	402 (13.1)	
60–69	1003 (19.4)	399 (19.1)	604 (19.7)	
70–79	1744 (33.8)	799 (38.2)	945 (30.8)	
≥ 80	1161 (22.5)	512 (24.5)	649 (21.2)	
Height, mean (SD), cm	159.2 (17.0)	158.7 (18.7)	159.5 (15.8)	0.104
Weight, mean (SD), kg	60.1 (13.7)	60.0 (13.8)	60.2 (13.7)	0.536
BMI, mean (SD), kg/m^2^	23.1 (4.4)	23.1 (4.5)	23.2 (4.4)	0.759
HF subtypes (*n* = 4968)				< 0.001
HFpEF (EF ≥ 50%)	1260 (25.4)	656 (32.6)	604 (20.4)	
HFmrEF(40% ≤ EF < 50%)	799 (16.1)	359 (17.9)	440 (14.9)	
HFrEF (EF < 40%)	2909 (58.6)	995 (49.5)	1914 (64.7)	
Comorbidity at baseline				
Hypertension	3228 (62.6)	1302 (62.3)	1926 (62.8)	0.699
Diabetes mellitus	2082 (40.4)	719 (34.4)	1363 (44.4)	< 0.001
Previous stroke	789 (15.3)	410 (19.6)	379 (12.4)	< 0.001
Previous chronic renal failure	732 (14.2)	259 (12.4)	473 (15.4)	0.002
IHD	2237 (43.4)	627 (30.0)	1610 (52.5)	< 0.001
VHD	991 (19.2)	608 (29.1)	383 (12.5)	< 0.001
Cardiomyopathy	1409 (27.3)	509 (24.3)	900 (29.3)	< 0.001
COPD	562 (10.9)	251 (12.0)	311 (10.1)	0.034
Medications before discharge				
Angiotensin-converting enzyme inhibitor	1980 (38.4)	714 (34.1)	1266 (41.3)	< 0.001
Angiotensin receptor blockers	2372 (46.0)	926 (44.3)	1446 (47.1)	0.043
Βeta-blockers	3012 (58.4)	1182 (56.5)	1830 (59.7)	0.025
Aldosterone antagonist	2888 (56.0)	1235 (59.1)	1653 (53.9)	0.001
Loop diuretics	4721 (91.5)	1965 (94.0)	2756 (89.9)	< 0.001
Digoxin	1708 (33.1)	1141 (54.6)	567 (18.5)	< 0.001
Warfarin	1669 (32.4)	1268 (60.6)	401 (13.1)	< 0.001
Aspirin	3362 (65.2)	1194 (57.1)	2168 (70.7)	< 0.001
Statin	2432 (47.2)	779 (37.3)	1653 (53.9)	< 0.001
Smoking				< 0.001
Current smoker	951 (18.4)	277 (13.2)	674 (22.0)	
Ex-smoker	1057 (20.5)	444 (21.2)	613 (20.0)	
Never smoker	3150 (61.1)	1370 (65.5)	1780 (58.0)	
Alcohol intake				0.288
Heavy alcoholic	356 (6.9)	153 (7.3)	203 (6.6)	
Social drinker	1623 (31.5)	635 (30.4)	988 (32.2)	
Never drinker	3179 (61.6)	1303 (62.3)	1876 (61.2)	

Data are reported as n (%)

*Abbreviations*: *AF* atrial fibrillation, *BMI* body mass index, *HF* heart failure, *EF* ejection fraction, *HFpEF* heart failure with preserved ejection fraction, *HFmrEF* heart failure with mid-range ejection fraction, *HFrEF* heart failure with reduced ejection fraction, *IHD* ischemic heart disease, *VHD* valvular heart disease, *COPD* chronic obstructive pulmonary disease

### Incidence rates for stroke and all-cause death after 1 and 2 years

During a mean follow-up of 2.3 years (up to 5.5 years), the rates of stroke in HF patients with and without AF were 7.0% (*n* = 147) and 4.1% (*n* = 125), respectively, and the rates of all-cause death were 38.7% (*n* = 807) and 37.2% (*n* = 1141), respectively (Additional file 1).

The overall incidence rates of stroke in the entire study cohort after 1 and 2 years were 4.4 and 3.0 per 100 person-years, respectively (Table 2). Incidence rates were generally attenuated (2.3/100 person-years) after 1 year of follow-up (Additional file 1), indicating that the majority of the events occurred relatively early after hospitalization for HF. Subgroup analyses showed that the incidence rates of stroke during the first year generally increased with increasing age. In patients with AF aged < 40 years and ≥ 80 years, the incidence rates of stroke were 2.6 and 8.6 per 100 person-years, respectively, whereas, in patients without AF, the incidence rates of stroke in these two age groups were 1.0 and 4.1 per 100 person-years, respectively. CHA_2_DS_2_-VASc scores of 1, 2, 3, 4, 5, 6, and ≥ 7 were associated with incidence rates of 5.3, 3.8, 3.2, 6.6, 5.7, 6.9, and 10.3 per 100 person-years, respectively, in patients with AF, and with incidence rates 4.0, 0.9, 4.0, 3.3, 3.8, 5.0, and 5.3 per 100 person-years, respectively, in patients without AF. Because patients with CHA_2_DS_2_-VASc scores of 1 were more likely to have cardiomyopathy than patients with CHA_2_DS_2_-VASc scores > 2, the high incidence rates of stroke in patients with CHA_2_DS_2_-VASc scores of 1 may result from underlying diseases such as cardiomyopathy (Additional file 2). Although the incidence rates of stroke were lower during the second year than the first year of follow-up, the incidence rates during the second year generally increased with increasing CHA_2_DS_2_-VASc score. As expected, the incidence of stroke was higher in patients with than without AF. The overall incidence rates of all-cause death in the entire study cohort after 1 and 2 years of follow-up were 26.2 and 20.5 per 100 person-years, respectively (Table 3). The incidence rate of all-cause death during the first year also generally increased with increasing age. In patients with AF aged < 40 and ≥ 80 years, the incidence rates of all-cause death were 10.1 and 43.6 per 100 person-years, respectively, whereas, in patients without AF, the incidence rates of all-cause death in these two age groups were 7.6 and 46.5 per 100 person-years, respectively. CHA_2_DS_2_-VASc scores of 1, 2, 3, 4, 5, 6, and ≥ 7 were associated with incidence rates of 14.6, 13.1, 22.1, 28.8, 28.0, 37.6, and 39.6 per 100 person-years, respectively, in patients with AF and with incidence rates of 11.0, 13.6, 17.6, 26.4, 32.7, 37.5, and 46.0 per 100 person-years, respectively, in patients without AF. The incidence rate of all-cause death was lower during the second than during the first year of follow-up, but it generally increased with increasing CHA_2_DS_2_-VASc score.

**Table 2:** Incidence rates of stroke at 1 and 2 year follow-up in the KorAHF study population, stratified according to prior diagnosis of atrial fibrillation

Characteristics	At 1 year follow-up				At 2 year follow-up			
	With AF (*n* = 2091)		Without AF (*n* = 3067)		With AF (*n* = 2091)		Without AF (*n* = 3067)	
	Patients (%)	IR	Patients (%)	IR	Patients (%)	IR	Patients (%)	IR
Overall	94 (4.5)	5.74	86 (2.8)	3.55	116 (5.5)	3.96	103 (3.4)	2.37
Age, years								
< 40	1 (2.2)	2.59	2 (0.9)	1.02	1 (2.2)	1.43	2 (0.9)	0.55
40–49	1 (1.0)	1.25	9 (3.7)	4.55	2 (2.0)	1.34	9 (3.7)	2.43
50–59	11 (4.7)	5.63	9 (2.2)	2.59	12 (5.1)	3.30	12 (3.0)	1.85
60–69	16 (4.0)	4.83	17 (2.8)	3.49	18 (4.5)	2.94	23 (3.8)	2.59
70–79	34 (4.3)	5.38	30 (3.2)	4.12	44 (5.5)	3.91	37 (3.9)	2.87
≥ 80	31 (6.1)	8.62	19 (2.9)	4.10	39 (7.6)	6.43	20 (3.1)	2.56
Sex								
Male	41 (3.8)	4.89	55 (3.3)	4.20	52 (4.9)	3.44	65 (3.9)	2.74
Female	53 (5.2)	6.63	31 (2.2)	2.79	64 (6.2)	4.53	38 (2.7)	1.93
CHA_2_DS_2_-VASc score								
1 (HF only)	6 (4.3)	5.29	7 (3.3)	4.00	7 (5.1)	3.32	7 (3.3)	2.13
2	9 (3.2)	3.79	4 (0.8)	0.93	9 (3.2)	2.03	5 (1.0)	0.62
3	9 (2.6)	3.19	16 (3.3)	4.04	14 (4.1)	2.69	18 (3.7)	2.47
4	21 (5.0)	6.61	14 (2.6)	3.26	21 (5.0)	3.71	21 (3.9)	2.73
5	18 (4.3)	5.66	17 (2.9)	3.82	22 (5.2)	3.90	20 (3.4)	2.56
6	14 (5.1)	6.91	16 (3.8)	5.04	22 (7.9)	6.36	17 (4.0)	3.07
≥ 7	17 (7.7)	10.27	12 (3.8)	5.32	21 (9.5)	7.65	15 (4.7)	3.91

Incidence rates per 100 person-years

*Abbreviations*: *AF* atrial fibrillation, *IR* incidence rate

**Table 3:** Incidence rates of death at 1 and 2 year follow-up in the KorAHF study population, stratified according to prior diagnosis of atrial fibrillation

Characteristics	At 1 year follow-up				At 2 year follow-up			
	With AF (*n* = 2091)		Without AF (*n* = 3067)		With AF (*n* = 2091)		Without AF (*n* = 3067)	
	Patients (%)	IR	Patients (%)	IR	Patients (%)	IR	Patients (%)	IR
Overall	446 (21.3)	26.45	643 (21.0)	26.05	626 (29.9)	20.61	905 (29.5)	20.36
Age, years								
< 40	4 (8.7)	10.09	15 (6.7)	7.57	5 (10.9)	6.98	20 (8.9)	5.42
40–49	16 (16.2)	19.82	30 (12.4)	14.67	17 (17.2)	11.21	37 (15.3)	9.63
50–59	30 (12.7)	14.89	41 (10.2)	11.63	41 (17.4)	10.91	68 (16.9)	10.30
60–69	56 (14.0)	16.45	111 (18.4)	22.28	91 (22.8)	14.41	153 (25.3)	16.80
70–79	177 (22.2)	27.23	226 (23.9)	30.44	241 (30.2)	20.59	321 (34.0)	24.30
≥ 80	163 (31.8)	43.59	220 (33.9)	46.52	231 (45.1)	36.28	306 (47.1)	38.31
Sex								
Male	230 (21.6)	26.77	354 (21.1)	26.41	316 (29.6)	20.24	500 (29.8)	20.51
Female	216 (21.1)	26.11	289 (20.8)	25.61	310 (30.2)	20.99	405 (29.1)	20.19
CHA_2_DS_2_-VASc score								
1 (HF only)	17 (12.3)	14.56	20 (9.5)	11.03	20 (14.5)	9.22	28 (13.3)	8.19
2	32 (11.5)	13.13	59 (11.7)	13.64	45 (16.1)	9.87	82 (16.2)	10.15
3	63 (18.5)	22.09	72 (14.8)	17.60	83 (24.3)	15.68	108 (22.1)	14.35
4	95 (22.8)	28.80	115 (21.3)	26.44	138 (33.2)	23.25	168 (31.1)	21.46
5	92 (21.9)	28.00	148 (25.5)	32.66	135 (32.1)	22.96	209 (36.0)	26.25
6	79 (28.5)	37.57	122 (28.8)	37.53	111 (40.1)	30.65	155 (36.6)	27.32
≥ 7	68 (30.9)	39.58	107 (33.8)	45.97	94 (42.7)	32.21	155 (48.9)	39.21

Incidence rates per 100 person-years

*Abbreviations*: *AF* atrial fibrillation, *IR* incidence rate

### Predictive accuracy of CHA_2_DS_2_-VASc score

At 1 year follow-up, each 1-point increase in CHA_2_DS_2_-VASc score, considered as a continuous variable, was associated with significantly increased risks of stroke in HF patients with (HR = 1.162, 95% CI = 1.028–1.313) and without (HR = 1.156, 95% CI = 1.006–1.328) AF, as well as significantly increased risks of all-cause death in HF patients with (HR = 1.165, 95% CI = 1.100–1.234) and without (HR = 1.213, 95% CI = 1.155–1.275) AF (Table 4). At 2 year follow-up, each 1-point increase in CHA_2_DS_2_-VASc score was also significantly associated with increased risk of stroke in HF patients with (HR = 1.237, 95% CI = 1.108–1.381) and without (HR = 1.144, 95% CI = 1.009–1.298) AF, and with significantly increased risk of all-cause death in HF patients with (HR = 1.192, 95% CI = 1.135–1.251) and without (HR = 1.216, 95% CI = 1.167–1.268) AF. The CHA_2_DS_2_-VASc score performed modestly in this population of patients with HF at 1 year follow-up. However, competing risk analysis showed that CHA_2_DS_2_-VASc score was not associated with increased risk of stroke in HF patients without AF. The C-indices for stroke in patients with and without AF were 0.598 (95% CI, 0.538–0.658) and 0.593 (95% CI, 0.534–0.652), respectively; and the C-indices for all-cause death in patients with and without AF were 0.600 (95% CI, 0.571–0.629) and 0.630 (95% CI, 0.606–0.653), respectively. The predictive ability after 2 years was slightly higher, but was still modest. The C-indices for stroke in patients with and without AF were 0.639 (95% CI, 0.585–0.694) and 0.613 (95% CI, 0.561–0.666), respectively; and the C-indices for all-cause death in patients with and without AF were 0.626 (95% CI, 0.600–0.652) and 0.635 (95% CI, 0.612–0.658), respectively. The predictive ability at endpoint in the KorAHF study population presented in Additional file 3.

**Table 4:** Assessment of the ability of CHA_2_DS_2_-VASc score to predict stroke and death at 1 and 2 year follow-up in the KorAHF study population, stratified according to prior diagnosis of atrial fibrillation

Characteristics	Overall (*n* = 5158)		With AF (*n* = 2091)		Without AF (*n* = 3067)	
	HR (95% CI)	*P*-value	HR (95% CI)	*P*-value	HR (95% CI)	*P*-value
At 1 year						
Stroke						
Model 1	1.157 (1.070–1.253)	< 0.001	1.165 (1.043–1.302)	0.007	1.145 (1.022–1.282)	0.019
Model 2	1.173 (1.072–1.283)	0.001	1.162 (1.028–1.313)	0.017	1.156 (1.006–1.328)	0.040
Model 3	1.151 (1.050–1.260)	0.003	1.140 (1.005–1.290)	0.042	1.145 (0.997–1.310)	0.054
C-index (95% CI)^a^	0.595 (0.536–0.654)		0.598 (0.538–0.658)		0.593 (0.534–0.652)	
Death						
Model 1	1.212 (1.174–1.251)	< 0.001	1.180 (1.121–1.242)	< 0.001	1.233 (1.183–1.284)	< 0.001
Model 2	1.196 (1.153–1.241)	< 0.001	1.165 (1.100–1.234)	< 0.001	1.213 (1.155–1.275)	< 0.001
C-index (95% CI)^a^	0.618 (0.599–0.636)		0.600 (0.571–0.629)		0.630 (0.606–0.653)	
At 2 year						
Stroke						
Model 1	1.187 (1.105–1.275)	< 0.001	1.212 (1.097–1.340)	< 0.001	1.157 (1.044–1.283)	0.006
Model 2	1.210 (1.116–1.313)	< 0.001	1.237 (1.108–1.381)	< 0.001	1.144 (1.009–1.298)	0.036
Model 3	1.181 (1.088–1.280)	< 0.001	1.204 (1.077–1.350)	0.001	1.128 (0.995–1.280)	0.061
C-index (95% CI)^a^	0.626 (0.573–0.680)		0.639 (0.585–0.694)		0.613 (0.561–0.666)	
Death						
Model 1	1.227 (1.194–1.260)	< 0.001	1.207 (1.156–1.260)	< 0.001	1.239 (1.197–1.283)	< 0.001
Model 2	1.210 (1.173–1.248)	< 0.001	1.192 (1.135–1.251)	< 0.001	1.216 (1.167–1.268)	< 0.001
C-index (95% CI)^a^	0.635 (0.612–0.658)		0.626 (0.600–0.652)		0.635 (0.612–0.658)	

Model 1: unadjusted model

Model 2: adjusted for previous chronic renal failure, ischemic heart disease, valvular heart disease, cardiomyopathy, chronic obstructive pulmonary disease (COPD), medications (Angiotensin-converting enzyme inhibitor, Angiotensin receptor blockers, Βeta-blockers, Aldosterone antagonist, Loop diuretics, Digoxin, Warfarin, Aspirin, Statin), and smoking

Model 3: competing risk model adjusted for previous chronic renal failure, ischemic heart disease, valvular heart disease, cardiomyopathy, chronic obstructive pulmonary disease (COPD), medications (Angiotensin-converting enzyme inhibitor, Angiotensin receptor blockers, Βeta-blockers, Aldosterone antagonist, Loop diuretics, Digoxin, Warfarin, Aspirin, Statin), and smoking after considering all-cause death as a competing risk

*Abbreviations*: *AF* atrial fibrillation, *HR* hazard ratio, *CI* confidence interval

^a^ From time-receiver operative characteristic (ROC) curve analysis

## Discussion

This study showed that CHA_2_DS_2_-VASc score was significantly associated with the risks of stroke and death in patients with HF, both with and without AF, and that CHA_2_DS_2_-VASc score was able to modestly predict the risk of stroke and death in these patients. This study also found that stroke mainly occurred during the early phase after hospitalization for HF. To our knowledge, this is the first study to evaluate the ability of CHA_2_DS_2_-VASc score to predict the risk of stroke and death in Asian patients with HF.

HF has a prevalence of approximately 1–2% among adult populations in developed countries, rising to ≥10% among individuals aged > 70 years [20]. In Korea, the prevalence of HF was 1.53% in 2013, and is expected to increase to 3.35% in 2040 [21]. In the present study, the 1 and 2 year all-cause mortality rates among patients with HF were 21.1 and 29.7%, respectively. By comparison, a previous study of individuals in the KorAHF registry reported that the 1, 2, and 3 year all-cause mortality rates were 18.2, 27.6, and 34.6%, respectively [15]. The discrepancy between these earlier results and ours is likely due, at least in part, to the exclusion from the present study of patients with a previous history of cancer. In addition, the present study population consisted of hospitalized patients with acute decompensation, a population with a relatively high mortality rate. For example, the European Society of Cardiology Heart Failure Pilot study showed that the 1 year all-cause mortality rates for hospitalized and stable/ambulatory HF patients were 17.4 and 7.2%, respectively [22], and a prospective observational cohort study in France reported that 20.8% of patients died within 1 year after the index hospitalization discharge [23]. HF-associated mortality rates have declined over the last 30 years, due to improvements in treatments and their implementation [20], but the mortality rate in patients with HF still remains high.

In the present study, the overall incidence rates of stroke during the first year after HF hospitalization in patients with and without AF were 4.5 and 2.8 per 100 person-years, respectively, rates higher than reported previously [12, 13]. For example, a nationwide prospective cohort study in Denmark reported that the incidence rates of ischemic stroke within 1 year after HF diagnosis were 2.0 and 1.0 per 100 person-years in patients with and without AF, respectively [12]. The difference may have been due to our inclusion of patients with more severe disease and/or to racial/ethnic differences in the risk of stroke. Indeed, among countries in the Organization for Economic Cooperation and Development (OECD), South Korea has a relatively high mortality rate from cerebrovascular diseases. Similar to the results from Denmark [12], we also found that the majority of strokes occurred relatively shortly after HF hospitalization, with 66.2% of all strokes occurring during the first year after HF hospitalization. Furthermore, the incidence rate of stroke during the first year after HF hospitalization was 3.5%, and the cumulative incidence during a mean follow-up of 2.3 years was 5.3%. These findings indicate that early intervention is essential for prevention of stroke in patients with HF.

Risk prediction models involving readily available clinical variables may be useful in preventing stroke and death in patients with HF. The CHA_2_DS_2_-VASc score [8] is an easily determined clinical risk score commonly used to stratify stroke risk in patients with AF [9]. The 2012 European Society of Cardiology (ESC) and the Korean Heart Rhythm Society (KHRS) guidelines recommend the use of CHA_2_DS_2_-VASc score to determine the necessity of treatment with an oral anticoagulant (OAC) or a non-vitamin K antagonist oral anticoagulant (NOAC) to prevent stroke [9, 24]. CHA_2_DS_2_-VASc score has been used to predict ischemic stroke, thromboembolism, and death in subjects without AF [10, 11] and in patients with incident HF with or without AF, although its predictive accuracy was modest (C-statistics 0.6–0.7) [12]. In addition, CHA_2_DS_2_-VASc score was reported to have only modest predictive accuracy for death and stroke in patients with systolic HF in sinus rhythm [13]. Similarly, a nationwide retrospective study of the Korea National Health Insurance Corporation sample cohort showed that the CHA_2_DS_2_-VASc score could help stratify stroke risk for individual HF patients (C-statistics 0.657), but its predictive ability was lower in patients with than without AF [25]. We found that CHA_2_DS_2_-VASc score, as a continuous variable, was able to modestly predict the risks of stroke and death in patients with HF with AF, and that CHA_2_DS_2_-VASc score was dependent on the length of follow-up. Although these initial results indicate the potential usefulness of CHA_2_DS_2_-VASc score, its direct clinical utility in stratifying stroke risk stratification in patients with HF remains unclear. Additional studies, testing the ability of a modified CHA_2_DS_2_-VASc score to predict the development of stroke and death in patients with HF, are needed.

This study had several limitations. First, patients with AF were included both recurrence and no recurrence patients, however we could not distinguish for these groups. Second, patients with stroke in this study included those with both ischemic and hemorrhagic stroke, as the sample size was too small to statistically analyze each outcome and stroke were not distinguished between ischemic and hemorrhagic stroke. Third, we could not evaluate the use of non-vitamin K antagonist oral anticoagulant (NOAC) therapy, because it have been reimbursed by the Korean National Health Insurance since 2015. Fourth, because the KorAHF registry enrolled patients who were hospitalized for acute HF, our results may have overestimated the predictive ability of the CHA_2_DS_2_-VASc score. Also, the CHA_2_DS_2_-VASc score might be more reasonably predictable in chronic stable HF patients compared to acute HF patients. Additional studies including patients with stable HF are required. However, this study reported risks 1 and 2 years after discharge, as well as after overall follow-up, suggesting that these results are comparable with the general HF population.

Despite these limitations, this study had several strengths. First, we used the KorAHF registry, a prospective, previously validated cohort study containing a large number of patients. Second, to our knowledge, this study is the first to investigate the risks of stroke and death, which are important when investigating the performance of risk scores in populations with a high mortality rate [26, 27], in patients with HF using CHA_2_DS_2_-VASc score. In addition, this study was the first to evaluate the ability of the CHA_2_DS_2_-VASc score to predict the risks of stroke and death in Asian patients with HF, with and without AF.

## Conclusions

Evaluation of patients in the KorAHF registry showed that the majority of strokes occurred relatively shortly after hospitalization for HF and that mortality rates in patients with HF remain high. Thus, early treatment after HF to prevent stroke is essential. CHA_2_DS_2_-VASc score was significantly associated with the risks of stroke in patients with acute HF patients with AF, and it was significantly associated with the risks of death in both HF patients with and without AF. Because CHA_2_DS_2_-VASc score had only a modest ability to predict the risk of stroke in these patients, the clinical utility of the CHA_2_DS_2_-VASc score in patients with HF remains to be determined. Future studies using a modified CHA_2_DS_2_-VASc score to predict the risks of stroke and death in patients with HF are needed.

## Additional files

Additional file 1: Incidence rates of stroke and death at endpoint in the KorAHF study population, stratified according to prior diagnosis of atrial fibrillation (PDF 130 kb)

Additional file 2: Frequency of cardiomyopathy according to baseline CHA_2_DS_2_-VASc score (PDF 44 kb)

Additional file 3: Assessment of the ability of CHA_2_DS_2_-VASc score to predict stroke and death at endpoint in the KorAHF study population, stratified according to prior diagnosis of atrial fibrillation (PDF 83 kb)

## Data Availability

Legal restrictions prohibit us from making our dataset publicly available. We were allowed to use the data from a South Korean government agency, Korea Centers for Disease Control (KCDC) on requested topics and we are not allowed to open the data to the public yet. The KorAHF registry is strictly managed by KCDC, which provides the data to researchers at the contribution of each institution to the cohort enrollment. Those who are interested in related data may contact and request the data from KCDC in South Korea (e-mail: nklim@korea.kr). The information of KorAHF registry can be obtained the following site: http://clinicaltrials.gov/ct2/show/NCT01389843

## Abbreviations

AF: Atrial fibrillation;
AUC: Area under the receiver operating characteristic curve
CI: Confidence interval
COPD: Chronic obstructive pulmonary disease
ESC: European society of cardiology
HF: Heart failure
HR: Hazard ratio
IHD: Ischemic heart disease
KHRS: Korean Heart Rhythm Society
KorAHF: The Korean acute heart failure
NOAC: Non-vitamin K antagonist oral anticoagulant
OAC: Oral anticoagulant
OECD: Organization for Economic Cooperation and Development
ROC: Receiver-operating characteristic
VHD: Valvular heart disease
