# Supplementary material for: Predicting stroke and death in patients with heart failure using CHA2DS2-VASc score in Asia
Source: BMC Cardiovasc Disord. 2019 Aug 8;19:193. doi: 10.1186/s12872-019-1178-0 (PMC6688312; doi:10.1186/s12872-019-1178-0)
Supplement: Supplementary file 1 — Incidence rates of stroke and death at endpoint in the KorAHF study population, stratified according to prior diagnosis of atrial fibrillation (PDF 130 kb) [file 12872_2019_1178_MOESM1_ESM.pdf]

Additional file 1. Incidence rates of stroke and death at end point in KorAHF study population, stratified according to prior diagnosis of atrial fibrillation

Characteristics	Overall (n=5,158)		With AF (n=2,091)		Without AF (n=3,067)	
	Cases (%)	IR	Cases (%)	IR	Cases (%)	IR
**Stroke**	272 (5.3)	2.30	147 (7.0)	3.13	125 (4.1)	1.75
**Age**, years						
< 40	3 (1.1)	0.40	1 (2.2)	0.88	2 (0.9)	0.32
40-49	12 (3.5)	1.30	2 (2.0)	0.75	10 (4.1)	1.53
50-59	29 (4.5)	1.65	15 (6.4)	2.37	14 (3.5)	1.25
60-69	52 (5.2)	2.03	25 (6.3)	2.38	27 (4.5)	1.79
70-79	113 (6.5)	2.94	64 (8.0)	3.62	49 (5.2)	2.36
≥ 80	63 (5.4)	3.13	40 (7.8)	4.63	23 (3.5)	2.00
**Sex**						
Male	140 (5.1)	2.19	64 (6.0)	2.61	76 (4.5)	1.93
Female	132 (5.5)	2.43	83 (8.1)	3.70	49 (3.5)	1.53
**CHA_2DS_2-VASc score**						
1 (HF only)	14 (4.0)	1.42	7 (5.1)	1.85	7 (3.3)	1.16
2	19 (2.4)	0.89	12 (4.3)	1.58	7 (1.4)	0.51
3	37 (4.5)	1.72	18 (5.3)	2.02	19 (3.9)	1.51
4	54 (5.6)	2.50	30 (7.2)	3.35	24 (4.4)	1.90
5	56 (5.6)	2.66	28 (6.7)	3.25	28 (4.8)	2.26
6	49 (7.0)	3.63	26 (9.4)	5.14	23 (5.4)	2.73
≥7	43 (8.0)	4.42	26 (11.8)	6.38	17 (5.4)	3.01
**Death**	1950 (37.8)	15.92	809 (38.7)	16.43	1141 (37.2)	15.58
**Age**, years						
< 40	32 (11.8)	4.22	8 (17.4)	6.80	24 (10.7)	3.75
40-49	61 (17.9)	6.41	18 (18.2)	6.59	43 (17.8)	6.34
50-59	136 (21.3)	7.55	50 (21.2)	7.64	86 (21.4)	7.50
60-69	313 (31.2)	11.81	115 (28.8)	10.55	198 (32.8)	12.70
70-79	725 (41.6)	18.08	326 (40.8)	17.37	399 (42.2)	18.71
≥ 80	683 (58.8)	32.84	292 (57.0)	32.03	391 (60.2)	33.48
**Sex**						
Male	1036 (37.8)	15.61	417 (39.1)	16.32	619 (36.9)	15.16
Female	914 (37.8)	16.29	392 (38.2)	16.55	522 (37.5)	16.09
**CHA_2DS_2-VASc score**						
1 (HF only)	58 (16.6)	5.70	23 (16.7)	5.87	35 (16.6)	5.59
2	160 (20.4)	7.39	58 (20.8)	7.42	102 (20.2)	7.38
3	243 (29.3)	10.99	108 (31.7)	11.86	135 (27.7)	10.39
4	407 (42.5)	18.11	193 (46.4)	20.30	214 (39.6)	16.50
5	439 (43.9)	20.10	174 (41.4)	19.15	265 (45.6)	20.79
6	341 (48.6)	24.32	138 (49.8)	25.70	203 (47.9)	23.46
≥7	302 (56.2)	29.47	115 (52.3)	25.91	187 (59.0)	32.19

Abbreviations: IR, incidence rate
Incidence rates per 100 person-years
