# Supplementary material for: Predicting stroke and death in patients with heart failure using CHA2DS2-VASc score in Asia
Source: BMC Cardiovasc Disord. 2019 Aug 8;19:193. doi: 10.1186/s12872-019-1178-0 (PMC6688312; doi:10.1186/s12872-019-1178-0)
Supplement: Supplementary file 2 — Frequency of cardiomyopathy according to baseline CHA2DS2-VASc score (PDF 44 kb) [file 12872_2019_1178_MOESM2_ESM.pdf]

Additional file 2. Assessment of CHA_2DS_2-VASc score for predicting of stroke and death at end point in the KorAHF study population, stratified according to prior diagnosis of atrial fibrillation

Characteristics	Overall (n=5,158)		With AF (n=2,091)		Without AF (n=3,067)	
	HR (95% CI)	*P*-value	HR (95% CI)	*P*-value	HR (95% CI)	*P*-value
**Stroke**						
Model 1	1.227 (1.150-1.308)	<0.001	1.238 (1.133-1.353)	<0.001	1.210 (1.102-1.329)	<0.001
Model 2	1.259 (1.171-1.354)	<0.001	1.279 (1.160-1.410)	<0.001	1.190 (1.062-1.334)	0.003
Model 3	1.203 (1.118-1.290)	<0.001	1.220 (1.105-1.350)	<0.001	1.149 (1.026-1.290)	0.016
C-index (95% CI)[a]	0.587 (0.553-0.622)		0.589 (0.540-0.637)		0.584 (0.536-0.633)	
**Death**						
Model 1	1.245 (1.216-1.275)	<0.001	1.220 (1.174-1.267)	<0.001	1.262 (1.223-1.302)	<0.001
Model 2	1.234 (1.200-1.269)	<0.001	1.211 (1.160-1.263)	<0.001	1.245 (1.199-1.292)	<0.001
C-index (95% CI)[a]	0.645 (0.629-0.660)		0.626 (0.602-0.650)		0.657 (0.637-0.676)	

Abbreviations: HR, hazard ratio; CI, confidence interval

Model 1: unadjusted model

Model 2: adjusted for previous chronic renal failure, ischemic heart disease, valvular heart disease, cardiomyopathy, COPD, medications and smoking.

Model 3: competing risk model adjusted for previous chronic renal failure, ischemic heart disease, valvular heart disease, cardiomyopathy, chronic obstructive pulmonary disease (COPD), medications, and smoking after considering all-cause death as a competing risk.

[a]Obtained using ROC curve analysis by non-parametric method
