# Supplementary material for: Predicting stroke and death in patients with heart failure using CHA2DS2-VASc score in Asia
Source: BMC Cardiovasc Disord. 2019 Aug 8;19:193. doi: 10.1186/s12872-019-1178-0 (PMC6688312; doi:10.1186/s12872-019-1178-0)
Supplement: Supplementary file 3 — Assessment of the ability of CHA2DS2-VASc score to predict stroke and death at endpoint in the KorAHF study population, stratified according to prior diagnosis of atrial fibrillation (PDF 83 kb) [file 12872_2019_1178_MOESM3_ESM.pdf]

Additional file 3. Frequency of cardiomyopathy according to the CHA_2DS_2-VASc score at baseline

**CHA_2DS_2-VASc score**	**Cardiomyopathy**	
	**Yes (n=1409)**	**No (n=3749)**
1	178(51.0)	171(49.0)
2	312(39.8)	472(60.2)
3	272(32.8)	557(67.2)
4	250(26.1)	707(73.9)
5	209(20.9)	792(79.1)
6	118(16.8)	583(83.2)
≥7	70(13.0)	467(87.0)

Data reported as n (%)
